# A Scoping Review of Trends in Atmospheric Pollution Research in Uganda (1990–2025)

**DOI:** 10.3390/toxics14070542

**Published:** 2026-06-23

**Authors:** Elizabeth Ainembabazi, Kim Young Hyun, Twalibu Nzanzu, Lee Cheol Min

**Affiliations:** 1 Department of Chemical and Environmental Engineering, Seokyeong University, Seoul 02713, Republic of Korea; rladudgus128@skuniv.ac.kr (K.Y.H.); cheolmin@skuniv.ac.kr (L.C.M.); 2 Department of Chemistry, Faculty of Science, Kyambogo University, Kampala P.O. Box 1, Uganda; ntwalibu@kyu.ac.ug

**Keywords:** Uganda, air quality, atmospheric pollutants, particulate matter, air pollution, emissions

## Abstract

Air pollution is an emerging environmental and public health concern in Uganda; however, the evolution of atmospheric pollution research in the country has not been comprehensively synthesized. This study presents a scoping review of peer-reviewed literature published between 1990 and 2025, examining the temporal trends in research output, key pollutants investigated, the study environments and research methodological approaches. A structured literature search was conducted across three academic databases (Google Scholar, Web of Science, and PubMed) and eligible studies were screened and analysed using a standardized data extraction framework. The results reveal highly uneven growth in research output, with minimal activity prior to 2010, followed by rapid expansion after 2015 and a pronounced surge between 2020 and 2025. Particulate matter (PM_2.5_ and PM_10_) dominated the literature across all periods, while gaseous pollutants such as NO_2_, SO_2_, CO, and O_3_ were comparatively underrepresented. Most studies were conducted in urban environments, particularly in Kampala, whereas rural ambient monitoring remained limited. Methodologically, the literature evolved from proxy-based and gravimetric approaches to the increased use of low-cost sensors, portable monitors and satellite-derived data. Despite recent advances, the predominance of short-term and spatially constrained studies highlights persistent gaps in long-term and nationally representative air quality monitoring. This review synthesizes trends, methodological developments, and evidence gaps in atmospheric pollution research in Uganda over a 35-year period, providing a foundation for strengthening future monitoring and policy frameworks.

## 1. Introduction

Air pollution is widely recognized as a major environmental determinant of the global disease burden, contributing to millions of premature deaths annually through cardiovascular, respiratory, and metabolic conditions [1,2]. Fine particulate matter (PM_2.5_) is of particular concern due to its ability to penetrate deep into the respiratory tract and enter the bloodstream, resulting in systemic health effects [3]. While the burden of air pollution is a global challenge, its impacts are disproportionately borne by low- and middle-income countries, where rapid urbanization, energy poverty, and limited regulatory capacity exacerbate exposure levels [1,2,4].

Sub-Saharan Africa has historically experienced limited air quality monitoring and research compared to other world regions, leading to substantial uncertainty in exposure estimates and health impact assessments [4]. However, recent advances in monitoring technologies, including satellite remote sensing and low-cost sensor networks, have begun to improve data availability across the region [5]. Uganda represents a critical case study within this context, characterized by rapid population growth, expanding urban centers, increasing vehicular traffic, and widespread reliance on biomass fuels for cooking and heating.

Since the mid-2000s, research on atmospheric pollutants in Uganda has expanded considerably. Early studies primarily focused on household air pollution associated with biomass fuel use and its effects on respiratory health, particularly among women and children [6,7]. More recent studies have shifted toward ambient air quality in urban environments, examining roadside pollution, spatial variability of particulate matter, and population-level exposure using both reference-grade instruments and calibrated low-cost sensors [5,8,9]. In addition, modelling and satellite-based approaches have been increasingly applied to compensate for sparse ground-based monitoring.

Despite this growing body of literature, the evidence base remains fragmented. The studies vary substantially in pollutants measured, monitoring duration, spatial coverage, and statistical reporting. Many studies report mean concentrations without accompanying measures of variability or sample sizes. This limits opportunities for quantitative synthesis and cross-study comparison. Furthermore, the research efforts are geographically concentrated in Kampala and surrounding urban areas, with comparatively limited representation of rural and peri-urban regions.

To date, no comprehensive review has examined long-term trends in atmospheric pollution research in Uganda, including changes in research focus, methodological approaches, and data quality over time. Addressing this gap is essential for identifying priority pollutants, methodological limitations, and evidence gaps that constrain effective air quality management and health risk assessment.

The aim of this review is, therefore, to synthesize trends in atmospheric pollution research conducted in Uganda between 1990 and 2025. Specifically, this scoping review seeks to characterize temporal trends in research output over the years, identify the pollutants and the environments most frequently studied, and examine the evolution of monitoring and analytical approaches.

By providing a scoping overview of three and a half decades of research, this study aims to inform future monitoring strategies, research priorities, and evidence-based air quality policies in Uganda. Unlike previous reviews that have focused on specific pollutants or health outcomes, this study provides a comprehensive temporal synthesis of atmospheric pollution research trends, methods, and evidence gaps in Uganda over a 35-year period.

## 2. Materials and Methods

A comprehensive literature search was conducted across multiple electronic databases, including Google Scholar, PubMed, and Web of Science. The search strategy combined keywords related to air pollution and Uganda using Boolean operators, search terms included ‘air quality’, ‘atmospheric pollutants’, ‘PM_2.5_’, ‘PM_10_’, ‘carbon monoxide (CO)’, ‘nitrogen dioxide (NO_2_)’, ‘ozone (O_3_)’, and ‘Uganda’. The search was restricted to English-language peer-reviewed studies published between January 1990 and December 2025. Additionally, the reference lists of eligible articles and relevant review papers were manually screened to identify further studies.

**Figure 1:**
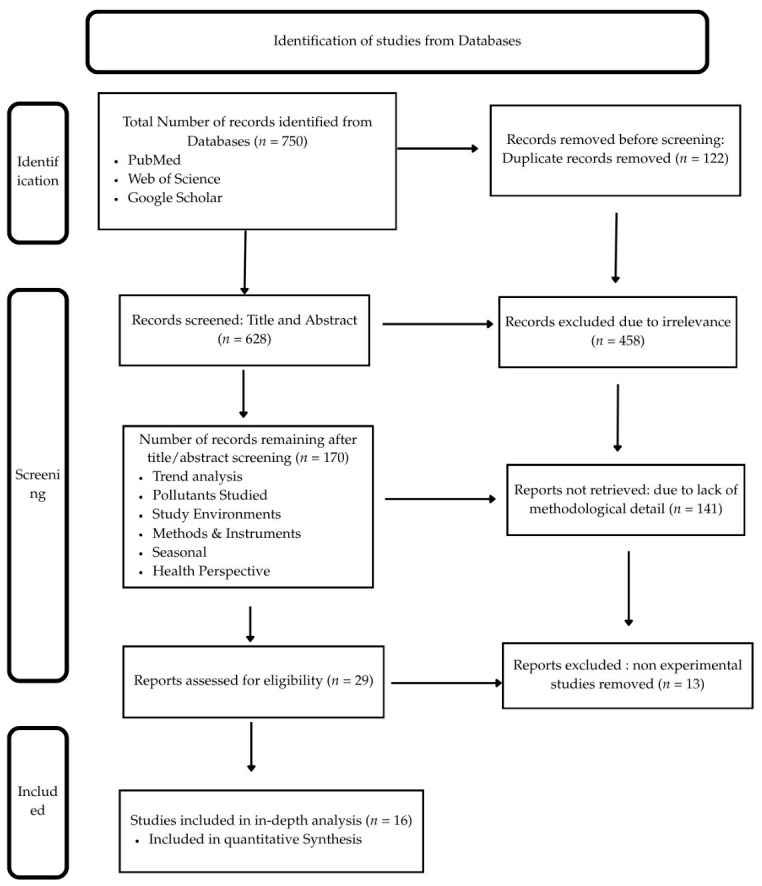
Flowchart of study identification, Screening and Inclusion.

During the systematic literature search, we identified a total of 750 records across Google Scholar, PubMed, and Web of Science. After removing the duplicates, 628 records remained for title and abstract screening. Of these, 458 papers were excluded due to irrelevance to air pollution, lack of empirical data, or focus on environmental media other than air (Figure 1).

A full-text assessment was conducted for 170 papers, resulting in the exclusion of 141 papers for reasons including a lack of Ugandan study sites, insufficient methodological detail, or the absence of atmospheric pollutant measurements. Ultimately, 29 studies met the eligibility criteria. Of these, 13 were non-experimental studies and were therefore excluded from the detailed qualitative synthesis. The remaining 16 experimental studies were included in the qualitative analysis. A subset of these studies provided sufficiently comparable quantitative data for consideration in descriptive statistical summaries, though formal meta-analysis was limited by inconsistent reporting of summary statistics.

The Inclusion and exclusion criteria used for study selection are summarized in (Table 1). Titles and abstracts were screened to exclude irrelevant records. The full texts of potentially eligible studies were then reviewed to confirm their inclusion.

For each included study, information was extracted on the publication year, study location, environment (ambient or indoor), study design, pollutants measured, monitoring or analytical methods, and key findings. Where available, summary statistics including mean concentrations, standard deviations, medians, and interquartile ranges were recorded, along with the sample size or number of monitoring sites.

The methodological quality of the included studies was assessed using adapted criteria for observational environmental studies. This assessment focused on the clarity of methods, appropriateness of measurement instruments, the duration of monitoring, and the transparency of data reporting.

A narrative synthesis was conducted to describe trends in the pollutants studied, methods used, and geographic coverage. A quantitative meta-analysis of pooled mean concentrations was initially considered but proved unfeasible for most studies due to inconsistent reporting of sample size (*n*)–means–standard deviations (SD) structures. Consequently, quantitative pooling was limited or not performed, and the findings are presented primarily as qualitative summaries.

The review was conducted in two stages. First, 170 studies that met the general relevance criteria were used to characterize overall trends in atmospheric pollution research in Uganda, including publication patterns, pollutants investigated, study environments, seasonal considerations, health perspectives, and methodological developments. Second, a subset of 16 studies selected from the 29 eligible studies was used for in-depth qualitative synthesis and descriptive quantitative analysis these 16 studies are included in Supplementary Table S1. This two-tier approach allowed for broad characterization of the research landscape while ensuring that detailed analyses were based on studies with comparable and adequately reported data. Section 3.1, Section 3.2, Section 3.3, Section 3.4, Section 3.5 and Section 3.6 are based on the 170 studies retained after title and abstract screening. Section 3.7 is based on the subset of 16 experimental studies selected for detailed synthesis.

## 3. Results

### 3.1. Trend of Studies on Atmospheric Pollutants in Uganda (1990–2025)

**Figure 2:**
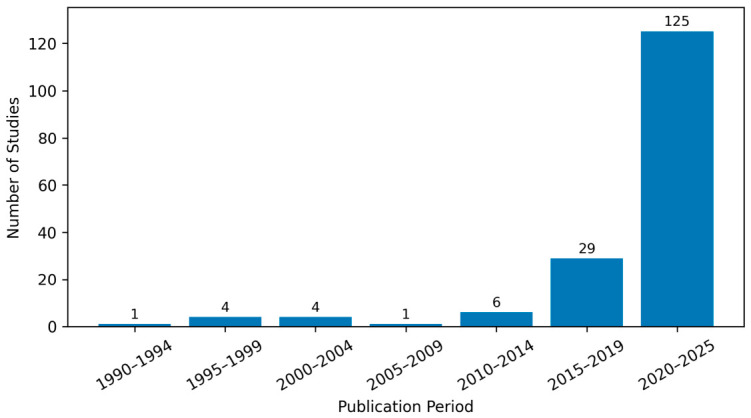
Number of air pollution studies over the years.

Analysis of the 170 screened records reveals a clear and highly uneven temporal distribution of studies on atmospheric pollutants in Uganda over the 1990–2025 period (Figure 2). Research output was extremely limited during the early years, with only one study published between 1990 and 1994, followed by four studies during 1995–1999 and another four studies between 2000 and 2004. This low level of activity reflects the absence of routine air quality monitoring infrastructure and limited research prioritization during this period. The period from 2005 to 2009 recorded only one study, indicating continued stagnation despite rapid urban growth. A gradual increase was observed between 2010 and 2014, with six studies published, coinciding with a growing awareness of household air pollution, traffic-related emissions, and associated health risks. A pronounced expansion in research activity occurred after 2014, with 29 studies published between 2015 and 2019, followed by a substantial surge to 125 studies between 2020 and 2025. This sharp increase aligns with the widespread adoption of low-cost air quality sensors, increased accessibility of satellite-based air pollution datasets, stronger international and regional research collaborations, and heightened policy and public health interest in air pollution in Uganda [5,10]. Despite this rapid growth, most studies conducted during the later periods remain short-term and geographically concentrated, underscoring persistent gaps in long-term, continuous monitoring and comprehensive national coverage.

### 3.2. Pollutants Studied

**Figure 3:**
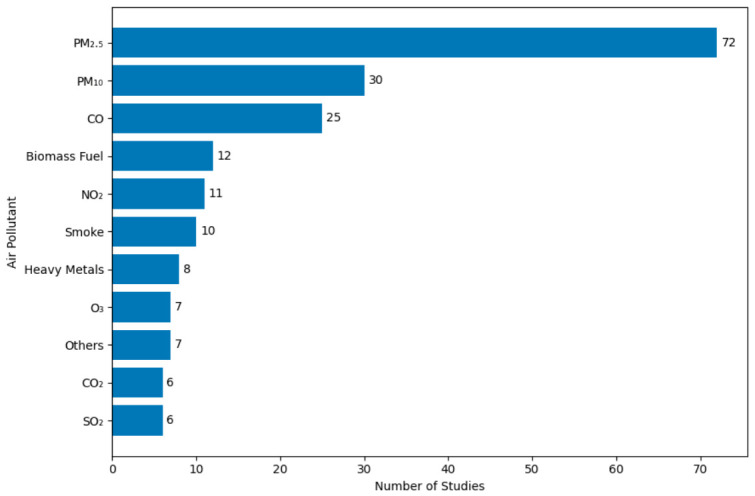
Distribution of Air Pollutants Investigated in Uganda (1990–2025).

Throughout the 1990–2025 period, the reviewed literature demonstrates a strong and persistent emphasis on particulate matter as the dominant class of atmospheric pollutants investigated in Uganda. Fine particulate matter (PM_2.5_) and coarse particulate matter (PM_10_) were the most frequently measured pollutants across both ambient outdoor and household environments. Within the included studies, PM_2.5_ was the most extensively investigated pollutant (n = 72), followed by PM_10_ (n = 30) and carbon monoxide (CO) (n = 25). Other pollutants received comparatively less attention, including nitrogen dioxide (NO_2_) (n = 11), heavy metals (n = 8), sulfur dioxide (SO_2_) (n = 6), and carbon dioxide (CO_2_) (n = 6) (Figure 3). In addition, several studies focused on pollution sources and exposure indicators such as biomass fuel use (n = 12) and smoke exposure (n = 10), while seven studies examined other pollutants or related atmospheric parameters. It is also important to note that several studies assessed multiple pollutants simultaneously.

Particulate matter measurements were predominantly conducted in urban environments, especially in Kampala, with roadside, traffic-influenced, and commercial areas consistently identified as pollution hotspots. In household settings, PM_2.5_ was widely used as an indicator of exposure to emissions from biomass fuel combustion, particularly charcoal and firewood. The reviewed studies reported that particulate matter concentrations frequently exceeded the World Health Organization (WHO) air quality guidelines, highlighting the severity of particulate pollution in both ambient and indoor environments.

In contrast, fewer studies were conducted on gaseous pollutants compared to particulate matter within the Ugandan air pollution literature. Nitrogen dioxide (NO_2_) and sulfur dioxide (SO_2_) were measured in a limited number of urban and roadside studies, primarily as indicators of vehicular and industrial emissions. Carbon monoxide (CO) was more commonly reported in household air pollution studies, where it served as a proxy for incomplete combustion resulting from solid fuel use. Ozone (O_3_) was the least studied pollutant, only a small number of studies assessed its concentrations or variability, often relying on satellite-derived or short-term measurements. Black carbon and other combustion-related pollutants were also infrequently assessed, despite their relevance to traffic emissions and climate forcing.

Overall, the distribution of investigated pollutants reveals a pronounced imbalance in the research focus, characterized by a strong dominance of particulate matter and the limited characterization of gaseous and secondary pollutants (such as ozone and secondary organic aerosols). While this focus aligns with the major public health burden associated with particulate exposure, it also underscores critical knowledge gaps related to comprehensive air quality assessments, atmospheric chemistry, and pollutant interactions in Uganda.

### 3.3. Study Environments

#### 3.3.1. Urban and Rural Study Settings

Studies conducted in urban environments overwhelmingly dominated the Ugandan air pollution literature between 1990 and 2025. Urban-focused investigations were primarily concentrated in Kampala, with additional studies conducted in other urban centers such as Jinja and Mbarara. These studies commonly targeted ambient particulate matter (PM_2.5_ and PM_10_), nitrogen dioxide (NO_2_), and, to a lesser extent, sulfur dioxide (SO_2_) and ozone (O_3_). Urban measurements were frequently conducted in roadside, commercial, and high-traffic locations, reflecting the influence of vehicular emissions, informal industrial activities, waste burning, and resuspended road dust. Across studies, urban pollutant concentrations were consistently higher than those reported in rural settings and frequently exceeded World Health Organization (WHO) air quality guidelines. Despite the high volume of urban studies, most relied on short-term monitoring campaigns, limiting their ability to capture long-term exposure trends.

In contrast, rural air pollution studies were comparatively limited in number and spatial coverage. Rural investigations were largely embedded within household air pollution research and focused primarily on emissions from biomass fuel use for cooking and heating. Ambient rural air quality measurements were relatively rare and typically involved short-term sampling periods. Where rural ambient data were available, pollutant concentrations were generally lower than in urban centers; however, episodic increases associated with agricultural burning, dust resuspension, and seasonal activities were reported. The underrepresentation of rural ambient studies highlights a significant gap in understanding background air pollution levels and rural exposure profiles in Uganda.

#### 3.3.2. Indoor, Outdoor and Personal Exposure Study Settings

**Figure 4:**
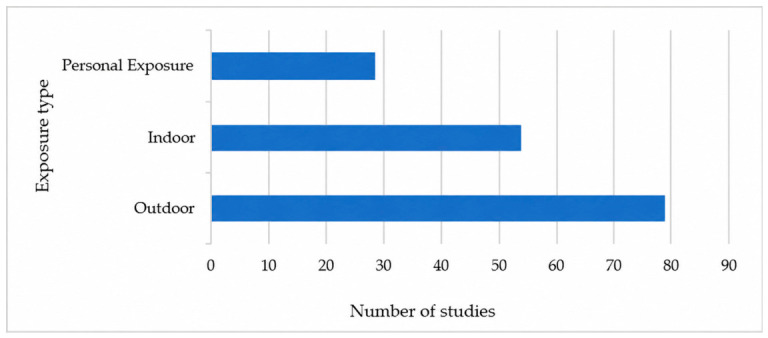
Distribution of Air Exposure study Types in Uganda (1990–2025).

Studies investigating air pollution exposure in Uganda were conducted in outdoor, indoor, and personal exposure settings, with outdoor studies constituting the largest proportion of the literature. As shown in Figure 4, outdoor studies were the most frequently reported, followed by indoor studies, whereas personal exposure studies represented the smallest category.

##### Outdoor Air Pollution Studies

Outdoor air pollution studies (n = 79) constituted the largest proportion of the reviewed literature. These studies primarily focused on ambient measurements in urban and peri-urban environments, with particulate matter (PM_2.5_ and PM_10_) serving as the dominant indicator pollutants. Monitoring locations commonly included roadside sites, residential neighborhoods, commercial areas, and institutional premises. Collectively, these investigations provided important insights into spatial variability, traffic-related emissions, and source influences. However, most outdoor studies were constrained by limited temporal coverage. Among the reviewed literature, studies featuring long-term continuous outdoor monitoring were rare.

##### Indoor Air Pollution Studies

Indoor air pollution studies (n = 53) represented the second largest category. These studies predominantly assessed exposure to particulate matter (PM_2.5_) and carbon monoxide (CO) within residential cooking environments. Measurements were commonly conducted in households relying on solid fuels such as charcoal, firewood, and agricultural residues, encompassing both urban and rural settings. Women and children were frequently identified as the most exposed populations due to the prolonged periods spent in cooking areas. Although some intervention-based studies evaluated improved cookstoves or ventilation strategies, the majority were cross-sectional and short-term in design, limiting the assessment of long-term exposure reductions and sustained intervention effectiveness.

##### Personal Exposure Studies

Personal exposure studies (n = 28) were the least represented category within the reviewed literature. These investigations typically employed portable monitoring devices to assess individual-level exposure to particulate matter and carbon monoxide (CO), often in conjunction with time–activity diaries. Personal exposure assessments were primarily conducted within household air pollution and occupational contexts. Findings frequently demonstrated weak correlations between individual exposures and fixed-site ambient measurements, underscoring the limitations of relying solely on stationary monitoring data for exposure estimation. The comparatively smaller number of personal exposure studies highlights a critical evidence gap, particularly given their importance in strengthening exposure–response analyses and accurately linking air pollution exposure to health outcomes.

### 3.4. Methods and Instruments

A wide range of monitoring approaches were identified. Early studies relied on gravimetric methods and short-term sampling campaigns, while more recent investigations have increasingly used continuous monitoring with reference grade instruments and calibrated low-cost sensors. Personal exposure monitoring was applied in only a limited number of health-focused studies. Further modelling and satellite-based approaches were also employed to supplement sparse ground measurements.

Based on the 170 studies that were retrieved, early studies conducted during the 1990s and early 2000s predominantly relied on indirect or proxy-based approaches. These included emission inventories, questionnaire-based exposure assessments, fuel-use surveys, and health or environmental proxies such as visibility and fuel consumption patterns. Direct ambient air quality measurements were rare during this period due to the limited availability of monitoring equipment and supporting laboratory infrastructure.

From approximately 2000 to 2010, a gradual transition toward direct measurement-based methods was observed. Studies during this period increasingly employed gravimetric sampling techniques to quantify particulate matter concentrations, particularly PM_10_ and total suspended particulates (TSP). These measurements were typically conducted using filter-based samplers deployed over short durations, often as part of cross-sectional field campaigns. Analytical methods during this phase focused on mass concentration determination, with limited chemical speciation or source apportionment.

Between 2010 and 2015, methodological diversification became more evident. Portable real-time monitoring instruments were increasingly adopted, allowing for higher temporal resolution measurements of particulate matter and selected gaseous pollutants. This period also marked the emergence of household air pollution exposure studies, which combined indoor monitoring with time–activity surveys to characterize personal exposure patterns. Statistical analyses during this phase remained largely descriptive, with limited application of advanced modelling or long-term trend analysis.

A pronounced methodological shift occurred after 2015, characterised by the widespread adoption of low-cost air quality sensors for measuring PM_2.5_ and PM_10_ in both ambient and household environments. These sensors enabled expanded spatial coverage, including dense urban monitoring networks and community-based measurements. Studies increasingly incorporated sensor calibration and validation exercises, often through co-location with reference-grade instruments. Concurrently, satellite-derived air pollution products and remote sensing techniques were frequently utilized to complement ground-based measurements and to assess spatial and temporal patterns at broader scales.

In the most recent period (2020–2025), research methods became more integrated and interdisciplinary. Studies increasingly combined ground-based monitoring, satellite observations, and meteorological data to assess pollutant variability and underlying drivers. Advanced analytical approaches, including spatial mapping, land-use regression, and exposure modelling, were more frequently applied. Nevertheless, most studies continued to rely on short-term monitoring campaigns, and relatively few employed continuous long-term measurements or longitudinal study designs.

### 3.5. Seasonal

Of the 170 screened papers, seasonal variability was inconsistently addressed across atmospheric pollution studies conducted in Uganda between 1990 and 2025. Among studies that explicitly reported seasonal context, measurements were commonly categorized according to Uganda’s bimodal climate, distinguishing between dry seasons (December–February and June–August) and wet seasons (March–May and September–November). However, a substantial proportion of the studies did not specify the season of data collection or were conducted over short monitoring periods that failed to capture seasonal transitions.

Where seasonal patterns were assessed, particulate matter concentrations—particularly PM_2.5_ concentrations were consistently higher during dry-seasons than during wet seasons. Elevated dry-season concentrations were attributed to increased resuspension of road dust, intensified biomass burning, reduced atmospheric dispersion, and limited wet deposition. Urban and roadside environments exhibited especially pronounced dry season increases, reflecting the combined influence of vehicular emissions and dust accumulation on unpaved or deteriorated road surfaces.

**Figure 5:**
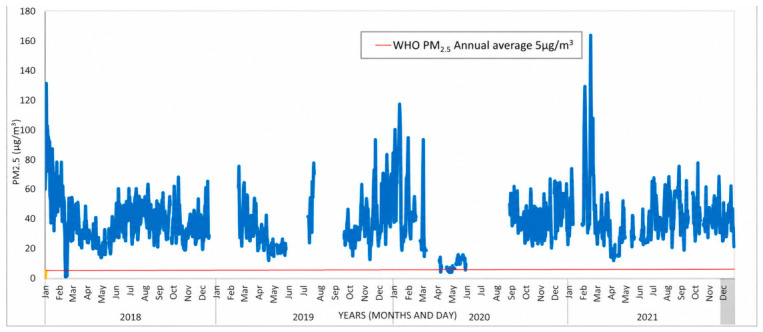
Seasonal variation in PM_2.5_ concentrations measured using a BAM 1022 monitor at the Makerere University School of Public Health, Kampala, Uganda (2018–2021). “Reproduced from [8]. Copyright 2025, Springer Nature”.

Evidence from continuous monitoring studies further supports these trends. For example, ref. [7] reported (Figure 5) PM_2.5_ concentrations frequently exceeded 80 µg/m^3^ during December, January, and February, with the highest recorded concentration occurring in February 2021, corresponding to the dry season. In contrast, the lowest PM_2.5_ concentrations were consistently observed in April and May, coinciding with peak rainfall. Across the 2018–2021 period, the reported annual mean PM_2.5_ concentration was 38.8 µg/m^3^ (SD = 18.6; range = 1.2–162.9 µg/m^3^), with an increasing trend from 2019 to 2021. This annual mean substantially exceeds the World Health Organization (WHO) 2021 annual PM_2.5_ guideline of 5 µg/m^3^ and frequently surpasses the 24 h guideline value of 15 µg/m^3^, indicating a persistent and significant public health concern [11,12].

Seasonal variation in gaseous pollutants was less frequently examined. Where reported, nitrogen dioxide (NO_2_) and carbon monoxide (CO) showed weaker and less consistent seasonal patterns than particulate matter, reflecting the dominance of local emission sources such as traffic and household combustion. Seasonal analysis of ozone (O_3_) was rare and often relied on satellite-derived estimates, highlighting a significant gap in understanding photochemical pollution dynamics in Uganda.

### 3.6. Health Perspective

The reviewed literature consistently demonstrates that air pollution poses a significant and growing public health challenge in Uganda. Across both ambient and household environments, exposure to elevated concentrations of particulate matter and combustion-related pollutants was associated with a range of adverse health outcomes, including respiratory symptoms, acute respiratory infections, reduced lung function, chronic respiratory disease, and premature mortality [6,8,13,14,15]. The health burden was not distributed equally across the population. Women and children under five years of age were repeatedly identified as highly exposed groups due to their prolonged presence in cooking environments where biomass fuels such as charcoal and firewood remain widely used [6,7]. Several studies reported elevated exposures to PM_2.5_ and CO in household settings, often exceeding international guideline values, thereby increasing the risk of respiratory morbidity and other pollution-related health effects [16,17,18]. Notably, a study conducted in Arua Municipality reported average PM_2.5_ and CO concentrations of 473 µg/m^3^ and 157 ppm, respectively, among households using fixed mud charcoal stoves, highlighting the severity of household air pollution exposure in some Ugandan settings [17].

Evidence from urban environments further highlighted the health implications of traffic-related particulate pollution and ambient air pollution. Studies conducted in Kampala and other urban centers reported associations between air pollution exposure and impaired respiratory health, particularly among children and other vulnerable populations [6,14,15]. More recently, continuous monitoring data from Kampala demonstrated that long-term exposure to elevated PM_2.5_ concentrations contributes substantially to attributable mortality among city residents [8]. These findings are consistent with global evidence linking particulate matter exposure to cardiovascular disease, respiratory disease, and premature death [1,2,3,12].

### 3.7. Quantitative Synthesis

Although several studies reported mean pollutant concentrations, very few provided the complete n–mean–SD structure required for pooled meta-analysis. Consequently, the quantitative synthesis of pooled mean concentrations was not feasible for most pollutants, and the findings are presented as descriptive summaries.

Across the extracted dataset from the 16 research reports included in the quantitative synthesis (Supplementary Table S1), PM_2.5_ had the largest number of measurable observations (n = 12) and exhibited substantial variability, with reported concentrations ranging from approximately 14.75 to 473 µg/m^3^. In comparison, fewer studies reported PM_10_ and CO (n = 4 each), However, it is important to note that some studies measured both and at times all the three parameters. Reported PM_10_ concentrations ranged from about 26 to 167 µg/m^3^, while CO concentrations ranged from approximately 28 to 157 ppm. The highest PM_2.5_ concentration (473 µg/m^3^) and CO concentration (157 ppm) were reported in households using fixed mud charcoal stoves in Arua Municipality, Uganda, where poor ventilation, prolonged cooking durations and inefficient stove design were identified as contributing factors [17]. These broad concentration ranges and high variability indicate marked heterogeneity in emission sources, monitoring environments, and measurement approaches across the studies (1990–2025) within the Ugandan evidence base.

The concentration ranges presented in Table 2 reflect measurements obtained under diverse monitoring conditions, including outdoor ambient environments, indoor household settings, and personal exposure assessments. Because these environments represent fundamentally different exposure scenarios, the reported ranges are intended to provide a descriptive overview of pollutant levels across the reviewed studies rather than direct quantitative comparisons.

## 4. Discussion

This review synthesizes 35 years (1990–2025) of research on atmospheric pollution in Uganda, demonstrating a clear growth in both the volume and scope of studies over time. The noticeable rise in publications after 2015 reflects the increasing recognition of air pollution as an important public health and environmental issue, coupled with improved access to monitoring technologies and stronger research collaborations [5,8,19]. While this growth has strengthened the national evidence base, the available literature remains concentrated around certain pollutants, locations, and study designs, which consequently shapes the current understanding of air quality in Uganda.

Particulate matter (PM_2.5_ and PM_10_) was the most frequently assessed pollutant across the reviewed studies. This focus is consistent with global evidence identifying fine particulate matter as a major contributor to cardiovascular and respiratory disease [1,2,3,12]. Across multiple studies, reported PM_2.5_ concentrations exceeded the WHO 2021 guideline values, in some cases substantially so. These findings reinforce the seriousness of particulate pollution as a public health concern in Uganda. The wide range of reported concentrations likely reflects differences in emission sources and monitoring environments, including traffic-related emissions, biomass combustion, industrial activity, open waste burning, and resuspended dust, particularly in rapidly urbanizing settings. While elevated particulate matter concentrations were frequently reported in roadside and urban commercial areas, some of the highest concentrations identified in this review were recorded in households relying on biomass fuels for cooking. For example, Muhwezi et al. reported average PM_2.5_ and carbon monoxide concentrations of 473 µg/m^3^ and 157 ppm, respectively, among households using fixed mud charcoal stoves in Arua Municipality [17]. These elevated concentrations were associated with poor ventilation, prolonged cooking durations, inefficient stove technologies, and confined cooking spaces. Similar findings from other household studies suggest that biomass fuel combustion remains an important contributor to population exposure, particularly among women and children who spend extended periods in cooking environments [6,7,16,18]. These findings highlight the need to address both ambient and household sources of pollution when developing air quality management and public health interventions in Uganda. Similar patterns have been reported across several Sub-Saharan African cities, including Nairobi (Kenya), Addis Ababa (Ethiopia), and Accra (Ghana), where PM_2.5_ concentrations frequently exceed WHO guideline values due to a combination of traffic emissions, biomass combustion, waste burning, and rapid urbanization. Similar to Uganda, these studies also report limited long-term monitoring infrastructure and substantial uncertainty in exposure assessment, highlighting a broader regional challenge in air quality management [4,20].

Compared to particulate matter, gaseous pollutants such as NO_2_, SO_2_, O_3_, and CO were less consistently monitored. As a result, the Ugandan evidence base is more heavily centered on particulate pollution, with relatively limited insight into broader atmospheric chemistry processes and secondary pollutant formation. This imbalance does not diminish the importance of particulate matter, but it does suggest that the overall pollutant profile remains only partially characterized. This trend may partly reflect the greater availability and affordability of particulate matter monitoring technologies, particularly low-cost PM sensors, compared with instruments required for measuring gaseous pollutants. Monitoring gases such as NO_2_, SO_2_, and O_3_ often requires more specialized equipment, calibration procedures, and maintenance, which may limit their inclusion in air quality studies conducted in resource-constrained settings [5]. Expanding the monitoring of gaseous pollutants would provide a more comprehensive understanding of air quality dynamics, pollutant interactions, and potential health risks in Uganda.

Geographically, most studies were conducted in Kampala and a few other urban centers. This urban focus reflects areas experiencing rapid population growth, increased vehicle density, and expanding commercial activity. Roadside and peri-urban monitoring frequently recorded elevated particulate concentrations, highlighting the influence of transport emissions and urban infrastructure conditions [5,9]. At the same time, household energy use remains a major component of Uganda’s air quality landscape. The widespread reliance on biomass fuels such as charcoal and firewood contributes significantly to indoor air pollution and subsequently affects ambient air quality [6,16,18]. Indoor and outdoor pollution sources are therefore closely interconnected, functioning as a unified environment rather than as discrete systems [16].

Methodologically, the increasing use of low-cost air quality sensors has expanded spatial coverage and enabled monitoring in previously data-scarce settings [5,19]. This has improved understanding of spatial variability, localized pollution hotspots, and air quality patterns across urban and peri-urban environments. However, these technologies also present several methodological challenges. Sensor performance may be influenced by environmental conditions such as humidity, temperature, and aerosol composition, and measurement accuracy often depends on calibration against reference-grade instruments [20]. In addition, sensor drift and maintenance requirements may introduce uncertainty during long-term monitoring campaigns. Although low-cost sensors have substantially expanded monitoring capacity in Uganda, long-term continuous monitoring using reference-grade instruments remains limited [5,8], constraining the evaluation of sustained multi-year trends. Consequently, because many studies relied on short-term sampling campaigns, the current evidence base remains skewed toward spatial differences and seasonal variability rather than long-term temporal patterns. Continued quality assurance, calibration procedures, and integration with reference monitoring systems will therefore be essential for ensuring reliable and comparable air quality measurements in the future [20].

Seasonal and meteorological conditions also appear to play an important role in shaping air pollution patterns in Uganda. Several studies reported higher particulate matter concentrations during the dry seasons compared to the wet seasons, reflecting the combined effects of increased dust resuspension, biomass burning, and reduced wet deposition [5,10,11]. Conversely, lower concentrations were generally observed during periods of higher rainfall, when precipitation promotes the removal of airborne particles from the atmosphere. Uganda experiences a bimodal rainfall regime characterized by major rainy seasons during March–May and September–November and relatively dry periods during December–February and June–August. Annual rainfall varies spatially across the country, ranging from approximately 750 to 2500 mm, with mean annual precipitation typically between 800 and 1500 mm. Peak rainfall generally occurs during April–May and October–November and is largely driven by the seasonal migration of the Inter-Tropical Convergence Zone (ITCZ) and associated regional circulation patterns [21]. These climatological conditions are consistent with the seasonal PM_2.5_ patterns observed across the reviewed studies, whereby enhanced wet deposition during rainy periods contributes to lower particulate matter concentrations while reduced precipitation during dry periods promotes pollutant accumulation [10,11]. These findings suggest that meteorological factors, including rainfall, atmospheric mixing, and seasonal emission patterns, should be considered when interpreting air quality measurements and designing monitoring strategies. Greater integration of meteorological data into future air quality studies would improve understanding of temporal variability and support more accurate exposure assessments.

Health-focused studies consistently linked air pollution exposure to respiratory symptoms and other adverse health outcomes, particularly among vulnerable populations such as women and children [6,7,13,14,15]. Despite increasing evidence of adverse health impacts, important knowledge gaps remain. Most Ugandan studies relied on cross-sectional designs and short-term exposure assessments, limiting the ability to establish causal relationships and evaluate long-term health effects. Furthermore, relatively few studies incorporated personal exposure monitoring, despite evidence that individual exposures often differ substantially from concentrations measured at fixed monitoring sites [7,15]. Strengthening longitudinal epidemiological research and integrating ambient, household, and personal exposure assessments will therefore be essential for improving understanding of the overall public health burden of air pollution in Uganda.

Overall, the evidence base reviewed here reflects a country undergoing rapid urbanization and energy transition, where air pollution is increasingly recognized as a pivotal environmental health issue. The consistent exceedance of WHO guideline values [12] (WHO, 2021) positions air quality at the forefront of broader national discussions on public health, urban development, transport systems, and household energy use. Addressing the identified gaps in long-term monitoring, rural air quality assessment, gaseous pollutant characterization, and exposure-based health research will be critical for strengthening the evidence base required to support effective air quality management and policy development in Uganda.

## 5. Conclusions

This scoping review demonstrates that atmospheric pollution research in Uganda has expanded substantially over the past 35 years, particularly after 2015, reflecting growing recognition of air pollution as an important environmental and public health challenge. The reviewed literature consistently identifies particulate matter (PM_2.5_ and PM_10_) as the most frequently studied pollutants and indicates that air pollution levels often exceed WHO guideline values in both urban outdoor environments and household settings reliant on biomass fuels.

Despite significant progress in monitoring technologies and research capacity, the current evidence base remains fragmented by differences in study design, exposure environments, monitoring approaches, and pollutants investigated. As a result, important gaps persist in long-term continuous monitoring, rural air quality assessment, gaseous pollutant characterization, and exposure-based health research. The predominance of short-term and cross-sectional studies also limits the ability to evaluate long-term trends and establish causal relationships between air pollution exposure and adverse health outcomes.

Nevertheless, the available evidence consistently suggests that air pollution represents an emerging environmental health burden in Uganda, with potential implications for respiratory health, cardiovascular disease, premature mortality, and broader public health outcomes. Strengthening integrated monitoring systems, expanding research beyond major urban centers, and improving the characterization of both ambient and household exposures will be critical for supporting evidence-based air quality management and public health policy in Uganda.

## Figures and Tables

**Table 1:** Inclusion and exclusion criteria.

Category	Criteria
Inclusion criteria	Conducted in Uganda. Focused on atmospheric or indoor air pollutants. Reported original quantitative data from monitoring, modelling, exposure assessment or epidemiological analysis. Included ambient outdoor and or indoor environments.
Exclusion Criteria	Focused solely on non-air media (e.g., water or soil without an air component). Studies on ultraviolet radiation or radionuclides. Biomonitoring of metals without corresponding air measurements. No quantitative pollutant concentration data. Policy, perception or awareness studies without exposure data.

**Table 2:** Summary of Reported Concentration Ranges of Key Air Pollutants in Uganda (1990–2025).

Pollutant	Number of Studies (n)	Reported Concentration Range
PM_2.5_ (µg/m^3^)	12	14.75–473
PM_10_ (µg/m^3^)	4	26–167
CO (ppm)	4	28–157

## Data Availability

No new data were created or analyzed in this study. Data sharing is not applicable to this article.

## Supplementary Materials

The following supporting information can be downloaded at https://www.mdpi.com/article/10.3390/toxics14070542/s1: Table S1: Studies included in the quantitative synthesis (n = 16).
