# Supplementary material for: A Scoping Review of Trends in Atmospheric Pollution Research in Uganda (1990–2025)
Source: Toxics. 2026 Jun 23;14(7):542. doi: 10.3390/toxics14070542 (PMC13416714; doi:10.3390/toxics14070542)
Supplement: Supplementary file 1 [file toxics-14-00542-s001.zip › toxics-4333352-supplementary.pdf]

Supplementary Table S 1: Studies included in the quantitative synthesis(n=16)

INCLUDED	SAMPLING METHOD	SD	MEAN	SAMPLE SIZE	ENVIRONMENT	YEAR	AUTHOR	TITLE
YES	Mixed-methods environmental assessment	NO	YES	yes	Urban ambient (Kampala)	2025	Deo Okure *a, Sarath K. Guttikunda *bc, Richard Sserunjogi a, Priscilla Adong a, Sai Krishna Dammalapati c, Dorothy Lsoto d, Paul Green a, Engineer Bainomugisha a and Jian Xie	Integrated air quality information for Kampala: analysis of PM2.5, emission sources, modelled contributions, and institutional framework
YES	Quantitative observational	YES	YES	YES	Rural household indoor kitchens	2020	N Nakora, D Byamugisha, G Birungi	Indoor air quality in rural Southwestern Uganda: particulate matter, heavy metals and carbon monoxide in kitchens using charcoal fuel in Mbarara Municipality
Yes	Quantitative observational	Yes	Yes	Yes	Urban ambient roadside	2021	A Singh, MJ Gatari, AW Kidane, ZA Alemu…	Air quality assessment in three East African cities using calibrated low-cost sensors with a focus on road-based hotspots
YES	Quantitative observational monitoring	yes	Yes	yes	Urban ambient (campus environment)	2021	EK Mukooza, S Kizza-Nkambwe	Effects of Lifting COVID-19 Lockdown on Ambient Air Particulate Matter and Associated Health Risk at Uganda Christian University's Main Campus, Mukono

yes	Field dust sampling ($PM_{2.5}$, PM_{10}), water samples lab analysis	yes	yes	yes	Outdoor ambient	2022	H Bakamwesiga, W Mugisha, Y Kisira, A Muwanga	An assessment of air and water pollution accrued from stone quarrying in Mukono District, Central Uganda
yes	Ground monitoring: US Embassy AQ, AirQo network	yes	yes	yes	Urban ambient	2020	T MUBARACK - 2020	Examining the effects of air pollution on health in Kampala City, Uganda
yes	24-h kitchen monitoring with particulate/CO sensors (field)	yes	yes	yes	Indoor (household cooking environment)	2025	G Muhwezi, D Ruhangariyo, J Kyayesimira	Quantification of fine particulate matter and carbon monoxide from households cooking with fixed mud charcoal stoves in Awindiri ward, Arua municipality, Uganda
yes	Laser particle detector ($PM_{2.5}$) and CO IAQ meter	yes	yes	yes	Informal urban settlement; mainly charcoal fuel use; ventilation and cooking location variables	2022	WK Kansiime, RK Mugambe, E Atusingwize	Use of biomass fuels predicts indoor particulate matter and carbon monoxide concentrations; evidence from an informal urban settlement in Fort Portal city, Uganda
condititional	Continuous ambient monitoring using BAM 1022 reference-grade instrument	yes	yes	yes	Urban outdoor environment	2024	Lynn M Atuyambe 1 2, Samuel Etajak 3 4, Felix Walyawula 5 3, Simon Kasasa 3 6, Agnes Nyabigambo 5 3, William Bazeyo 3, Heather Wipfli 7, Jonathan M Samet 8, Kiros T Berhane	Air quality and attributable mortality among city dwellers in Kampala, Uganda: results from 4 years of continuous PM2.5 concentration monitoring using BAM 1022 reference instrument

yes	Prospective cohort study	yes	yes	yes	Rural households,	2019	Crystal M North 1 2 3, Piers MacNaughton 4, Peggy S Lai 5 4 6, Jose Vallarino 4, Samson Okello 4 7 8, Bernard Kakuhikire 7, Alexander C Tsai 5 6 7, Marcia C Castro 4, Mark J Siedner 5 6 7, Joseph G Allen 4 6, David C Christiani 5 4	Personal carbon monoxide exposure, respiratory symptoms, and the potentially modifying roles of sex and HIV infection in rural Uganda: a cohort study
yes	Environmental monitoring / observational	yes	yes	yes	Urban and peri-urban area	2022	Richard Sserunjogi 1, Joel Ssematimba 1, Deo Okure 1, Daniel Ogenrwot 1, Priscilla Adong 1, Lillian Muyama 1, Noah Nsimbe 1, Martin Bbaale 1, Engineer Bainomugisha 1	Seeing the air in detail: Hyperlocal air quality dataset collected from spatially distributed AirQo network
yes	Environmental monitoring / observational study	yes	yes	yes	Urban, peri-urban, and rural sites	2019	Silver Onyango 1, Beth Parks 2, Simon Anguma 3, Qingyu Meng 4	Spatio-Temporal Variation in the Concentration of Inhalable Particulate Matter (PM10) in Uganda
yes	Comparative environmental monitoring study	yes	yes	yes	Institutional kitchens (schools, hospitals), urban Uganda	2017	A I McCord 1 2 3, S A Stefanos 1 3, V Tumwesige 3 4 5, D Lsoto 3, A H Meding 1, A Adong 3, J J Schauer 6, R A Larson 1	The impact of biogas and fuelwood use on institutional kitchen air quality in Kampala, Uganda
yes	Pilot cross-sectional spatial assessment	yes	yes	yes	Urban outdoor environment	2015	Bruce J Kirenga 1, Qingyu Meng 2, Frederik van Gemert 3, Hellen Aanyu-Tukamuhebwa 4, Niels Chavannes 5, Achilles Katamba 6, Gerald Obai 7, Thys van der	The State of Ambient Air Quality in Two Ugandan Cities: A Pilot Cross-Sectional Spatial Assessment

								Molen 8, Stephan Schwander 9, Vahid Mohsenin	
yes	Observational exposure study (cross-sectional / cohort)	yes	yes		yes	Household indoor environment (rural and peri-urban)	2018	Gabriel Okello 1, Graham Devereux 2, Sean Semple 3	Women and girls in resource poor countries experience much greater exposure to household air pollutants than men: Results from Uganda and Ethiopia
yes	Environmental sampling & laboratory analysis	yes	yes		yes	outdoor environment	2023	M Opolot, T Omara, C Adaku, E Ntambi	Pollution Status, Source Appointment, Ecological and Human health risks of potentially (Eco) toxic element-laden dusts from urban roads, highways and pedestrian bridges in Uganda
